# Retraction: MiR-144 Inhibits Uveal Melanoma Cell Proliferation and Invasion by Regulating c-Met Expression

**DOI:** 10.1371/journal.pone.0274144

**Published:** 2022-08-31

**Authors:** The PLOS ONE Editors

Following the publication of this article [1], similarities were noted between this article and articles submitted by other research groups, including [2–6], of which several were previously retracted [7–10].

Similarities included the following figures, which appear to fully or partially overlap, despite being published in different articles and representing different conditions:

- The control panel in Fig 2C of [1] flipped horizontally and vertically, and the Scramble panel in the corrected Fig 3C of [2,11].
- The c-Met panel in Fig 4A of [1] flipped horizontally and vertically, and lanes 2 and 3 of the PAQR3 panel in Fig 5C of [3,7].

The corresponding author stated that their article [1] may be similar to others [2–6] because they believe their data may have been used by others without their knowledge or permission, as it was not stored securely. They provided data files which do not resolve the concerns regarding similarities between the published articles [1–6].

The unresolved concerns call into question the validity and provenance of the reported results, and the adherence of this article to the PLOS Authorship policy. Therefore, the *PLOS ONE* Editors retract this article [1].

SM did not agree with the retraction. All other authors either did not respond directly or could not be reached.
